# Supplementary material for: Comparison of the human gastric microbiota in hypochlorhydric states arising as a result of Helicobacter pylori-induced atrophic gastritis, autoimmune atrophic gastritis and proton pump inhibitor use
Source: PLoS Pathog. 2017 Nov 2;13(11):e1006653. doi: 10.1371/journal.ppat.1006653 (PMC5667734; doi:10.1371/journal.ppat.1006653)
Supplement: S1 Text — (DOCX) [file ppat.1006653.s001.docx]

**Supplementary Methods**

**Mock Bacterial Community**

A mock bacterial community was used to evaluate the V1-V2 primers and monitor run-to-run variability. It consisted of RNA extracted from pure cultures of *Salmonella enterica* Typhimurium LT2, *Escherichia coli* K12, *Shigella sonnei,* *Helicobacter felis* ATCC 49179, *Proteus mirabilis*, *Streptococcus pyogenes* M1, *Staphylococcus aures, Burkholderia dolosa* BCC232, and *Pseudomonas aeruginosa* (kindly provided by Profs Barry Campbell and Craig Winstanley, University of Liverpool). V1-V2 primers showed good coverage, but data from mock communities suggested that these primers were unable to differentiate the closely related *E. coli*, *S. sonnei* and *S. enterica*. Furthermore, these three genera were often attributed to the genus *Tannerella* during analysis of gastric samples.

**Sequence analysis**

Samples initially underwent base-calling and de-multiplexing using CASAVA 1.8.2 (Illumina) before Illumina adapter sequences were removed using Cutadapt 1.2.1 [1]. Low quality bases were then removed using Sickle 1.200, with reads shorter than 10bp removed. The analysis was performed using the QIIME software package [2]. Paired-end reads were assembled using FLASH 1.2.8 [3] and samples were compared to Genbank (GenBank gi9626372) using BLASTn [4] to identify and subsequently remove PhiX sequences. Only samples within the expected V1-V2 range (min length 300, max length 400) were included in further analysis.

Qiime version 1.8.0 [2] was used for metagenomic analysis. The usearch quality filter pipeline [5] was used to filter putative chimeras and to pick Operational Taxonomic Units (OTUs). Sequences were clustered at 97% to reduce sequencing and PCR errors. Taxonomies were assigned using Qiime 1.8.0 with a RDP classifier [6] to match sequences to the Greengenes 12.8 database [7]. Relationships between OTUs were calculated by generating phylogeny based on Pynast [2] and were generated using the fasttree algorithm [8].

Tax4Fun [9] was used to predict the functional capabilities of microbial communities based on 16S rRNA datasets after blasting the OTUs against silva database (all prokaryotic KEGG organisms are available in Tax4Fun for SILVA SSU Ref NR database release 123 and KEGG database release 64.0) and then utilising ultrafast protein classification (UProC) tool [10] to generate metabolic functional profiles after normalising the data for 16S rRNA gene copy numbers.

**Statistical analysis**

Statistical analyses were performed in R using the tables and data generated as above as well as the meta data associated with the study. For community analysis (including alpha and beta diversity analyses) the Vegan [11] package was used. To calculate Unifrac distances (that account for phylogenetic closeness), the phyloseq [12] package was used. R's aov() was used to calculate pair-wise ANOVA p-values which were then drawn on top of alpha diversity figures. Nonmetric Distance Scaling (NMDS) plot of community data (OTUs at 3% divergence) using different distance measures (Vegan ’s metamds() function): BrayCurtis considers the species abundance count; Unweighted Unifrac considers the phylogenetic distance between the branch lengths of OTUs observed in different samples without taking into account the abundances; The samples are grouped for different conditions as well as the mean ordination value and spread of points (standard deviations of the (weighted) averages as ellipse using Vegan ’s ordiellipse() function).

To understand multivariate homogeneity of groups dispersions (variances) between multiple conditions, Vegan's betadisper() function was used as a multivariate analogue of Levene's test for homogeneity of variances. Non-euclidean distances between objects and group centroids were handled by reducing the original distances (BrayCurtis or Unweighted Unifrac) to principal coordinates and then performing ANOVA on them. Vegan's adonis() was used for analysis of variance using distance matrices (BrayCurtis/Unweighted Unifrac) i.e., partitioning distance matrices among sources of variation (both qualitative information, disease type, time points etc, and quantitative information, Gastrin, BMI etc.). This function, hitherto referred to as PERMANOVA, fits linear models (e.g. factors, polynomial regression) to distance matrices and uses a permutation test with pseudo-F ratios.

To find OTUs/Genera that were significantly different between different conditions, the DESeqDataSetFromMatrix() function was used from the DESeq2 [13] package with a significance value cut-off of 0.001. This function allows negative binomial GLM fitting (as abundance data from metagenomic sequencing is overdispersed) and Wald statistics for abundance data. After performing multiple testing corrections, it reports OTUs/Genera that have log-fold changes between multiple conditions. This method along with beta diversity analysis was repeated after the removal of *H. pylori* from all samples to ensure the dominance of this bacterium did not skew the proportions of the other bacteria in a misrepresentational way. This was done by removing the *H. pylori* OTUs from the abundance table and then performing differential expression analysis on the remaining data on the raw abundances.

For pathways analysis, i.e., to find KEGG K numbers significantly up/down regulated between multiple conditions, the Kruskal-Wallis test was used with p-values and multiple testing correction (Bonferonni Hochberg) p-adjusted values. Since Tax4Fun returned around 6K KEGG K numbers (proportional representation within a sample), and significant KEGG K numbers are around 1K or more, it was not possible to draw all of them in a single figure without introducing cluttering, so as a result only the top 200 most significantly different KEGG K numbers were drawn. Next, at most 358 pathways were considered (map/ko) available for meteganomes from gageData [14] package in R (database is kegg.sets.ko). The 50 most changed pathways (where the majority of the KEGG K numbers were found to be different) were included. R's pathview package [15] was utilised, which downloads KEGG pathways and colours the images (by drawing heatmaps on top of these images) by splitting KEGG K number/ EC number boxes into two boxes (for pair-wise comparison). To be able to distinguish subtle KEGG K number changes, the data were square-root transformed and the average KEGG K values for each condition with the red colour representing the maximum possible value of K numbers were plotted i.e. sqrt(maximum(K value)) across all the samples for each pair-wise comparison).

For generating co-occurence networks and subcommunity analysis, methods from Williams et al (2014) were followed [16]. Once subcommunities on network were obtained, network statistics such as Centrality, Betweenness, Closeness, and Eigenvector centrality were calculated (https://en.wikipedia.org/wiki/Centrality) which can identify keystone species (i.e., subcommunities where Eigenvector centrality is low and Betweenness is high). Network analysis was done using R packages igraph [http://igraph.org], sna [17], and network [18]. The statistical scripts and workflows for all above can be found at http://userweb.eng.gla.ac.uk/umer.ijaz#bioinformatics.

Differences between groups for serum gastrin concentrations, number of OTUs, and numbers of OTU observations were performed in GraphPad Prism and SPSS v 22. Samples were checked for equality of variance and for a normal distribution using Levene’s test of equality, the Kolmogorov-Smirnov test and the D’Agostino & Pearson normality test, prior to Kruskal-Wallis with Dunn’s post hoc test for serum gastrin concentrations, and 1-way ANOVA and Tukey’s multiple comparison test for OTUs.

**SUPPLEMENTARY REFERENCES**

1 Martin M. Cutadapt removes adapter sequences from high-throughput sequencing reads. EMBnetjournal 2011;**17**:10-2.

2 Caporaso JG, Kuczynski J, Stombaugh J, Bittinger K, Bushman FD, Costello EK*, et al.* QIIME allows analysis of high-throughput community sequencing data. Nature methods 2010;**7**:335-6.

3 Magoc T, Salzberg SL. FLASH: fast length adjustment of short reads to improve genome assemblies. Bioinformatics 2011;**27**:2957-63.

4 Altschul SF, Gish W, Miller W, Myers EW, Lipman DJ. Basic local alignment search tool. J Mol Biol 1990;**215**:403-10.

5 Edgar RC. Search and clustering orders of magnitude faster than BLAST. Bioinformatics 2010;**26**:2460-1.

6 Wang Q, Garrity GM, Tiedje JM, Cole JR. Naive Bayesian classifier for rapid assignment of rRNA sequences into the new bacterial taxonomy. Applied and environmental microbiology 2007;**73**:5261-7.

7 McDonald D, Price MN, Goodrich J, Nawrocki EP, DeSantis TZ, Probst A*, et al.* An improved Greengenes taxonomy with explicit ranks for ecological and evolutionary analyses of bacteria and archaea. The ISME journal 2012;**6**:610-8.

8 Price MN, Dehal PS, Arkin AP. FastTree: computing large minimum evolution trees with profiles instead of a distance matrix. Mol Biol Evol 2009;**26**:1641-50.

9 Asshauer KP, Wemheuer B, Daniel R, Meinicke P. Tax4Fun: predicting functional profiles from metagenomic 16S rRNA data. Bioinformatics 2015;**31**:2882-4.

10 Meinicke P. UProC: tools for ultra-fast protein domain classification. Bioinformatics 2015;**31**:1382-8.

11 Oksanen J, Blanchet FG, Kindt R, Legendre P, Minchin PR, O'Hara RB*, et al.* vegan: Community Ecology Package, R Package version 2.2-1 <http://CRAN.R-project.org/package=vegan>. 2015.

12 McMurdie PJ, Holmes S. phyloseq: an R package for reproducible interactive analysis and graphics of microbiome census data. PloS one 2013;**8**:e61217.

13 Love MI, Huber W, Anders S. Moderated estimation of fold change and dispersion for RNA-seq data with DESeq2. Genome Biol 2014;**15**:550.

14 Luo W. gageData: Auxillary data for gage package. R package version 2.8.0. 2013.

15 Luo W, Brouwer C. Pathview: an R/Bioconductor package for pathway-based data integration and visualization. Bioinformatics 2013;**29**:1830-1.

16 Williams RJ, Howe A, Hofmockel KS. Demonstrating microbial co-occurrence pattern analyses within and between ecosystems. Front Microbiol 2014;**5**:358.

17 Butts CT. Social Network Analysis with sna. Journal of Statistical Software, 24(6). ​<http://www.jstatsoft.org/v24/i06/>. 2008.

18 Butts CT. network: A Package for Managing Relational Data in R. Journal of Statistical Software, 24(2). 2008.
