# Supplementary figures and images for: Comparison of the human gastric microbiota in hypochlorhydric states arising as a result of Helicobacter pylori-induced atrophic gastritis, autoimmune atrophic gastritis and proton pump inhibitor use
Source: PLoS Pathog. 2017 Nov 2;13(11):e1006653. doi: 10.1371/journal.ppat.1006653 (PMC5667734; doi:10.1371/journal.ppat.1006653)

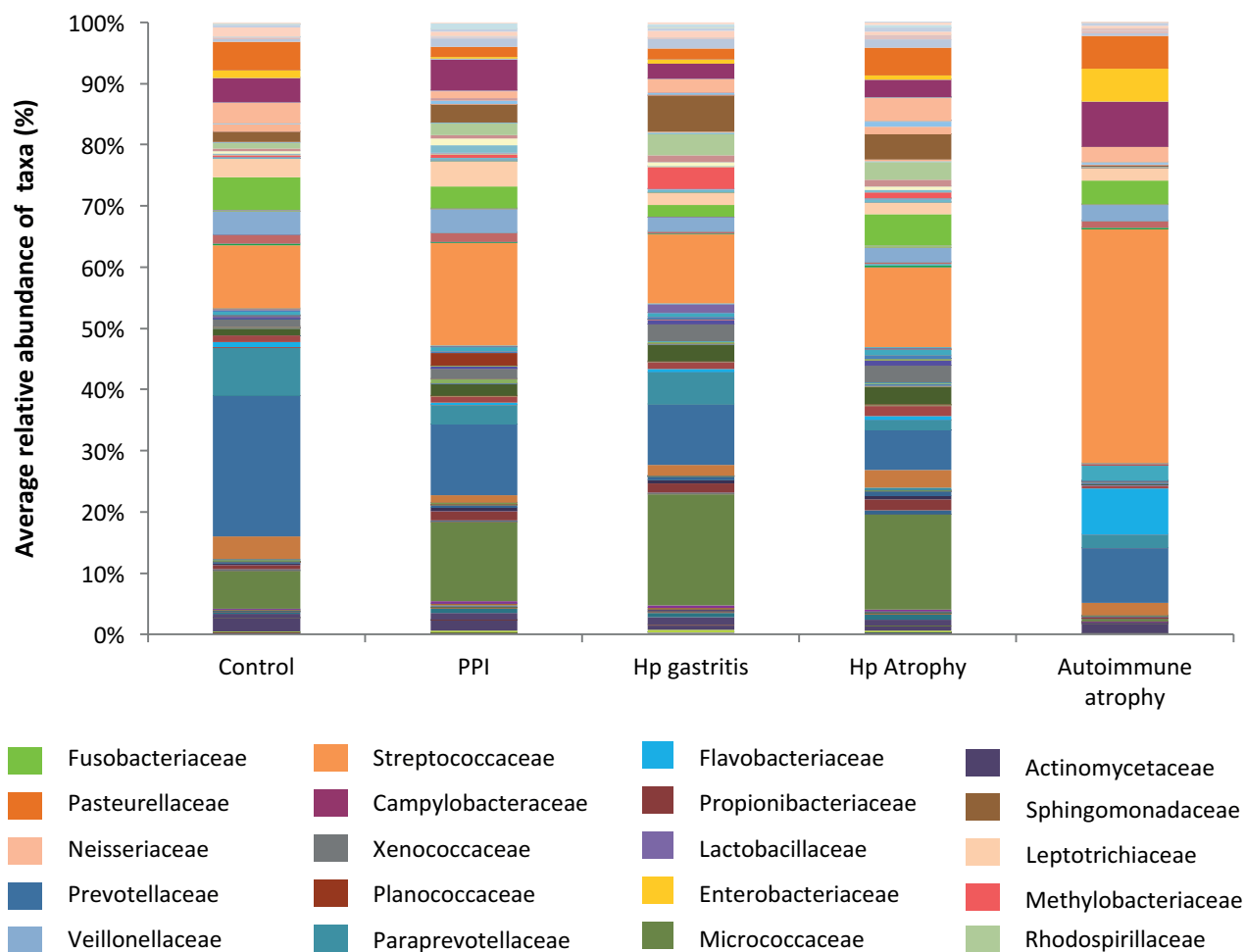

Supplement: S1 Fig — Hp = H. pylori, IM = intestinal metaplasia, IM+At = intestinal metaplasia and atrophy, PPI = proton pump inhibitor. All H. pylori atrophy samples were positive for H. pylori by serology. (PDF) [file ppat.1006653.s002.pdf]

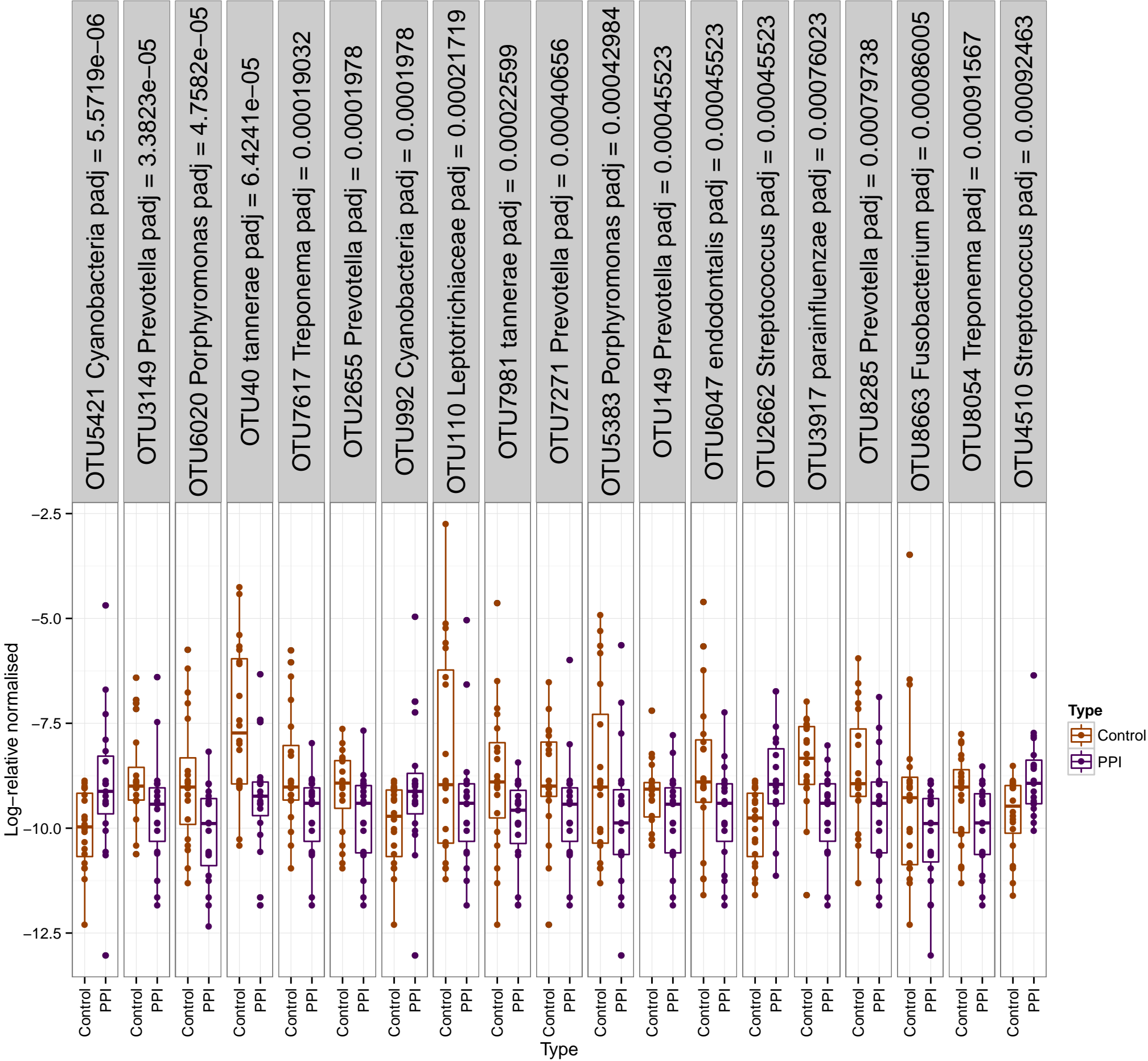

Supplement: S2 Fig — The most significant OTUs are identified on the left. Differential expression analysis based on the Negative Binomial (Gamma-Poisson) distribution. (PDF) [file ppat.1006653.s003.pdf]

**A**

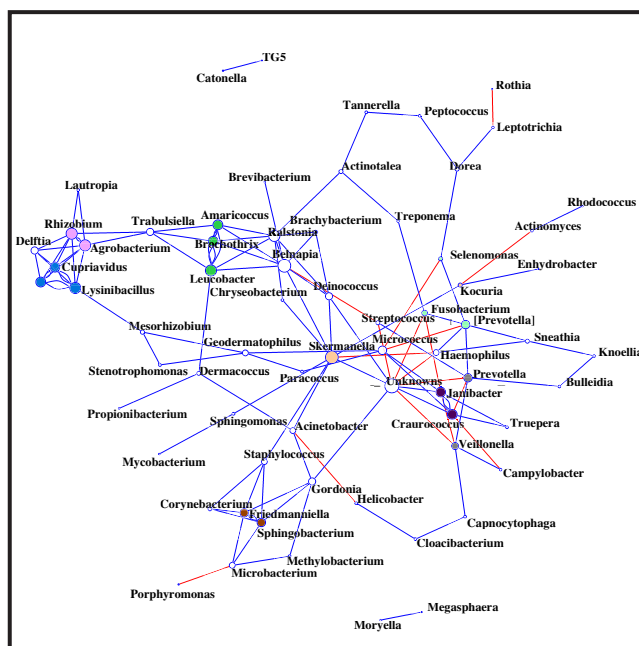

**PPI**

**B**

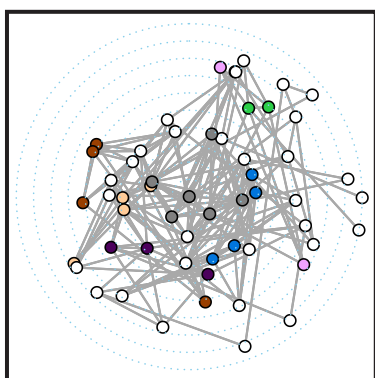

**Degree**

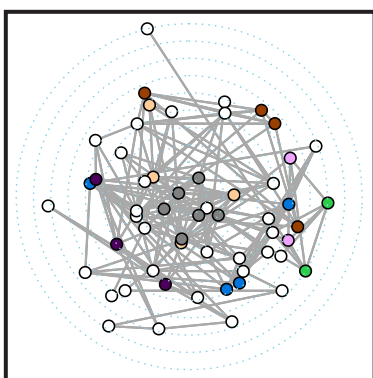

**Closeness**

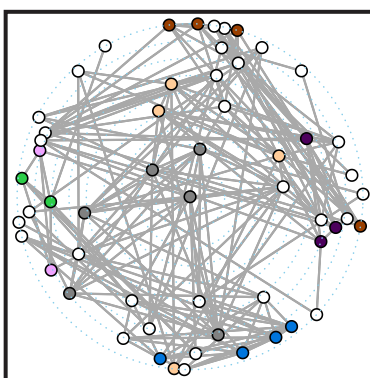

**Betweenness**

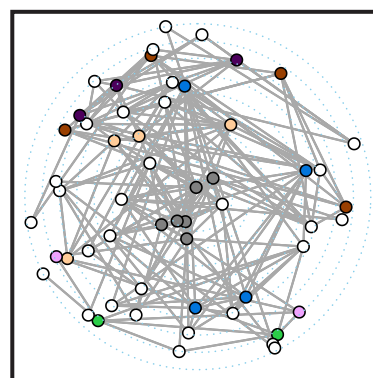

**Eigenvector**

Supplement: S3 Fig — Co-occurrence network analysis between different genera (OTUs collated together at genus level) when considering samples for (A) PPI. The genera were connected (Blue: positive correlation; Red: negative correlation) when the pair-wise correlation values were significant (P.adj<0.05) after adjusting the P values for multiple comparisons. Furthermore, subcommunity detection was performed by placing the genera in the same subcommunity (represented by colour of nodes) when many links were found at correlation values >0.75 between members of the subcommunity. The size of the nodes represent the degree of connections (B) network-wide statistics by degree, closeness, betweenness and eigenvalue centrality for H. pylori atrophic gastritis cases. The nodes (coloured with respect to subcommunity they are part of) were placed on concentric circles with values increasing from center to the periphery. A high betweenness for a node suggests many connections, whereas a high eigenvalue centrality suggests that those connections, in turn, are all well connected. On average a high betweenness and at the same time low eigenvalue centrality for a subcommunity suggests a keystone/important subcommunity. (PDF) [file ppat.1006653.s004.pdf]

**A****Antrum**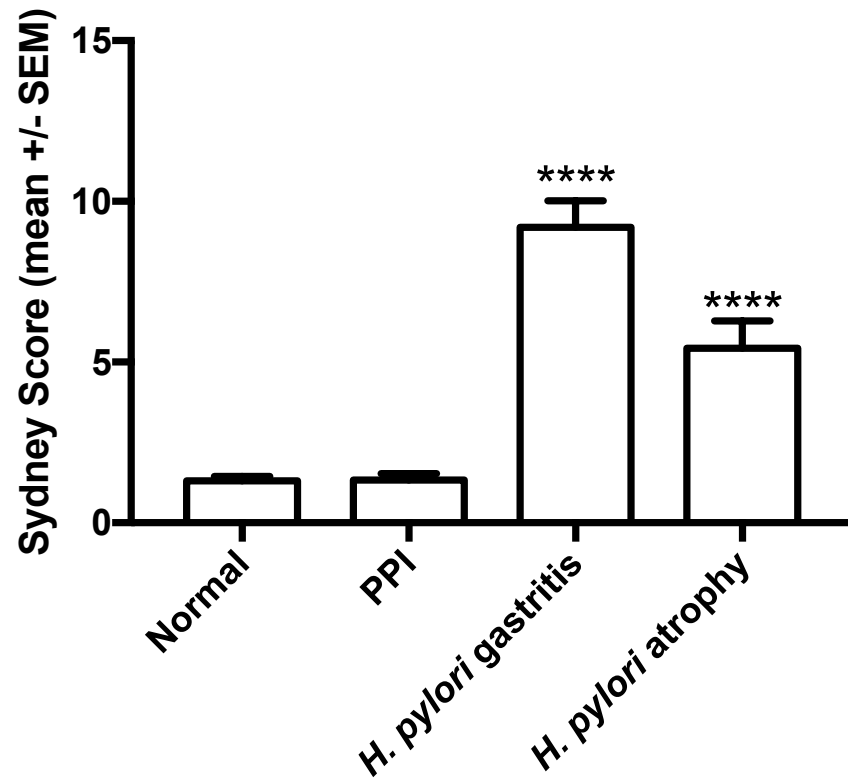**B****Corpus**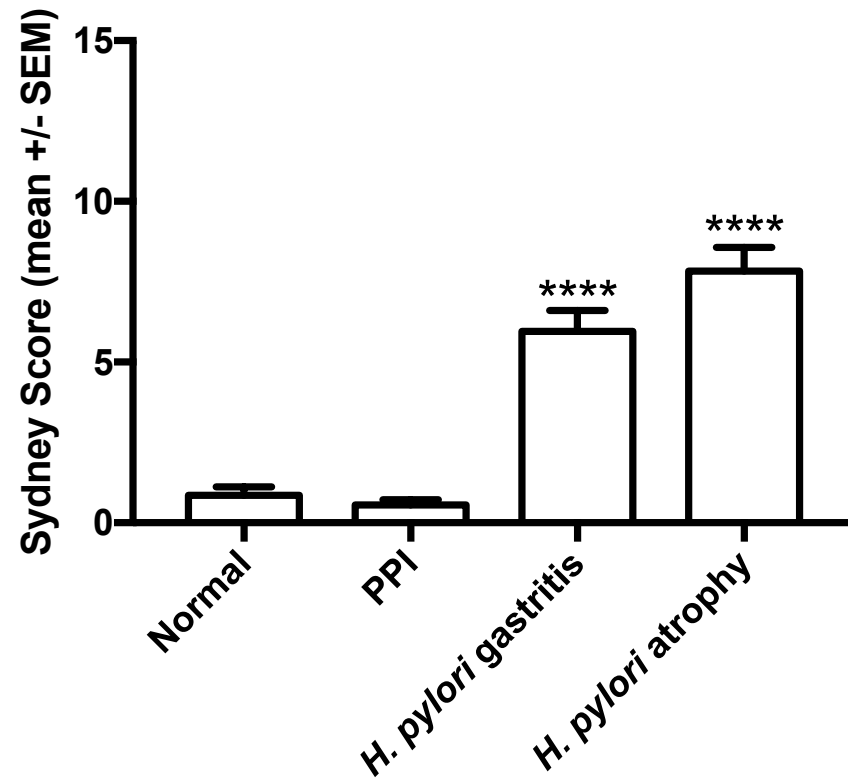

Supplement: S4 Fig — Updated Sydney scores were the sum of the scores for 5 individual parameters each scored 0–3 [40]. **** p<0.0001 versus control from the same mucosal site by 2-way ANOVA using Dunnett’s multiple comparison test. (PDF) [file ppat.1006653.s005.pdf]
