# Supplementary material for: Comparison of the human gastric microbiota in hypochlorhydric states arising as a result of Helicobacter pylori-induced atrophic gastritis, autoimmune atrophic gastritis and proton pump inhibitor use
Source: PLoS Pathog. 2017 Nov 2;13(11):e1006653. doi: 10.1371/journal.ppat.1006653 (PMC5667734; doi:10.1371/journal.ppat.1006653)
Supplement: S1 Table — The most significant species are identified at the top. Differential expression analysis based on the Negative Binomial (Gamma-Poisson) distribution and were corrected for multiple comparisons. * indicates a genus no longer significant when H. pylori was removed from the analysis. (DOCX) [file ppat.1006653.s006.docx]

**Table S1.** Significantly different genera identified between normal stomach samples and other patient groups

| **Comparison** | | **Genus** | **baseMean** | **log2 Fold Change** | **P value** | **P value adjusted** | **Upregulated** |
| --- | --- | --- | --- | --- | --- | --- | --- |
| Control | PPI | Actinobacillus | 48.14508705 | -2.74090110 | 2.00E-06 | 0.000347806 | Control |
|  |  | Tannerella | 119.161677 | -2.47237173 | 9.94E-06 | 0.000864436 | Control |
|  | Hp Gastritis | Helicobacter | 87634.15509 | 6.994269191 | 6.25E-26 | 8.68E-24 | HPGast* |
|  |  | Fusobacterium | 1159.653633 | -4.66927217 | 1.67E-15 | 7.84E-14 | Control |
|  |  | Selenomonas | 70.012747 | -4.43529494 | 1.69E-15 | 7.84E-14 | Control |
|  |  | Treponema | 285.1369457 | -4.65243841 | 1.01E-13 | 3.52E-12 | Control |
|  |  | Tannerella | 100.0995947 | -4.43575524 | 2.69E-13 | 7.47E-12 | Control |
|  |  | Actinomyces | 296.235288 | -4.17766654 | 3.75E-13 | 8.68E-12 | Control |
|  |  | Bulleidia | 20.89667007 | -3.65442646 | 2.20E-12 | 4.38E-11 | Control |
|  |  | Prevotella | 4439.258121 | -4.25503093 | 3.58E-12 | 6.22E-11 | Control |
|  |  | Haemophilus | 350.1633746 | -3.81079904 | 5.15E-12 | 7.95E-11 | Control |
|  |  | Veillonella | 479.0081536 | -3.92458686 | 5.90E-11 | 8.21E-10 | Control |
|  |  | Lautropia | 53.82120791 | -3.94243248 | 6.54E-11 | 8.26E-10 | Control |
|  |  | Parvimonas | 16.68299988 | -3.44902546 | 7.95E-11 | 9.21E-10 | Control |
|  |  | Porphyromonas | 584.6737119 | -4.07270167 | 1.49E-10 | 1.59E-09 | Control |
|  |  | Leptotrichia | 258.6870681 | -3.87373803 | 4.53E-10 | 4.50E-09 | Control |
|  |  | Streptococcus | 1890.180867 | -3.55716311 | 1.30E-09 | 1.21E-08 | Control |
|  |  | Aggregatibacter | 65.00318326 | -3.69848299 | 2.26E-09 | 1.97E-08 | Control |
|  |  | Dialister | 13.72121362 | -3.34705219 | 2.83E-09 | 2.31E-08 | Control |
|  |  | Trabulsiella | 14.48201526 | -3.28338822 | 5.14E-09 | 3.97E-08 | Control |
|  |  | Catonella | 17.58169996 | -3.38226142 | 1.16E-08 | 8.46E-08 | Control |
|  |  | Granulicatella | 28.16244662 | -3.27287378 | 1.22E-08 | 8.50E-08 | Control |
|  |  | Prevotella | 1517.78851 | -3.70892434 | 1.49E-08 | 9.88E-08 | Control |
|  |  | Campylobacter | 477.7806931 | -3.14511511 | 7.44E-08 | 4.70E-07 | Control |
|  |  | Actinobacillus | 43.80398687 | -3.55266753 | 8.74E-08 | 5.06E-07 | Control |
|  |  | Moryella | 17.09582821 | -3.15924527 | 8.65E-08 | 5.06E-07 | Control |
|  |  | Capnocytophaga | 121.3674756 | -3.20260682 | 1.11E-07 | 6.15E-07 | Control |
|  |  | Paludibacter | 21.24295418 | -3.21815177 | 1.39E-07 | 7.45E-07 | Control |
|  |  | Neisseria | 408.5284781 | -3.26565801 | 1.55E-07 | 7.99E-07 | Control |
|  |  | Atopobium | 5.925453204 | -2.50624750 | 4.18E-07 | 2.08E-06 | Control |
|  |  | Dorea | 7.25320838 | -2.62457551 | 8.08E-07 | 3.87E-06 | Control |
|  |  | Kingella | 9.399453182 | -2.88687294 | 9.23E-07 | 4.28E-06 | Control |
|  |  | Delftia | 8.970039432 | -2.52311262 | 1.07E-06 | 4.81E-06 | Control |
|  |  | Peptococcus | 4.362116597 | -2.42798849 | 1.41E-06 | 6.14E-06 | Control |
|  |  | Stenotrophomonas | 7.43364594 | -2.43816334 | 1.53E-06 | 6.44E-06 | Control |
|  |  | Filifactor | 13.81465948 | -2.65166442 | 4.68E-06 | 1.91E-05 | Control |
|  |  | Oribacterium | 5.982239935 | -2.16603316 | 8.16E-06 | 3.24E-05 | Control |
|  |  | Paracoccus | 25.36502287 | -2.6872075 | 8.81E-06 | 3.40E-05 | Control |
|  |  | Mycoplasma | 10.66803122 | -2.48760663 | 1.30E-05 | 4.89E-05 | Control |
|  |  | Rhodococcus | 10.28638726 | -2.26694688 | 1.70E-05 | 6.20E-05 | Control |
|  |  | Butyrivibrio | 3.658862276 | -2.19606704 | 1.79E-05 | 6.37E-05 | Control |
|  |  | Megasphaera | 21.26512058 | -2.27241891 | 0.000105335 | 0.000366038 | Control |
|  |  | Rothia | 49.23235848 | -2.42121854 | 0.000119191 | 0.000404085 | Control |
|  |  | Acinetobacter | 20.07140129 | -2.17601129 | 0.000137514 | 0.000455105 | Control |
|  |  | Unknowns | 2490.384423 | -2.37355056 | 0.000161354 | 0.000521587 | Control |
|  |  | Propionibacterium | 41.9659198 | -2.33178411 | 0.000179131 | 0.000565892 | Control |
|  |  | Schwartzia | 2.728662977 | -1.70806985 | 0.000258324 | 0.000797934 | Control |
|  |  | Microbacterium | 25.5890564 | -2.37695666 | 0.000273054 | 0.000825099 | Control |
|  |  | Pseudonocardia | 2.798220709 | -1.81476027 | 0.000285093 | 0.000843148 | Control |
|  |  | Pseudomonas | 11.97968985 | -2.05601315 | 0.000306857 | 0.000888607 | Control* |
|  | Hp Atrophy | Tannerella | 99.47059438 | 4.18313149 | 6.99E-14 | 1.02E-11 | Control |
|  |  | Helicobacter | 50674.75084 | -4.97220212 | 1.28E-13 | 1.02E-11 | Atrophy* |
|  |  | Treponema | 284.5929033 | 4.15280513 | 2.41E-12 | 1.27E-10 | Control |
|  |  | Selenomonas | 71.47054708 | 3.642026561 | 4.66E-12 | 1.85E-10 | Control |
|  |  | Mycoplasma | 10.99412303 | 2.910436462 | 2.57E-08 | 6.81E-07 | Control |
|  |  | Catonella | 17.57419555 | 3.031599688 | 2.28E-08 | 6.81E-07 | Control |
|  |  | Prevotella | 1504.971458 | 3.294863507 | 7.70E-08 | 1.75E-06 | Control |
|  |  | Peptococcus | 4.29719532 | 2.460544765 | 1.89E-07 | 3.75E-06 | Control |
|  |  | Bulleidia | 22.32141703 | 2.483869117 | 1.83E-06 | 3.23E-05 | Control |
|  |  | Butyrivibrio | 3.572387352 | 2.231982069 | 2.63E-06 | 3.79E-05 | Control |
|  |  | Pseudomonas | 11.33992081 | 2.348254597 | 2.56E-06 | 3.79E-05 | Control |
|  |  | TG5 | 57.03395136 | 2.67407275 | 5.29E-06 | 7.02E-05 | Control |
|  |  | Aggregatibacter | 71.02444579 | 2.600319806 | 1.64E-05 | 0.000201056 | Control |
|  |  | Fusobacterium | 1251.661271 | 2.646368629 | 1.80E-05 | 0.000204541 | Control |
|  |  | Prevotella | 4737.349827 | 2.602414793 | 2.77E-05 | 0.000293252 | Control |
|  |  | Leptotrichia | 277.0643808 | 2.518473005 | 3.00E-05 | 0.000297702 | Control |
|  |  | Dialister | 14.67947296 | 2.248561093 | 3.98E-05 | 0.000372279 | Control |
|  |  | Abiotrophia | 3.046547717 | 1.961344161 | 5.78E-05 | 0.000498271 | Control |
|  |  | Actinomyces | 321.6194581 | 2.420155942 | 5.95E-05 | 0.000498271 | Control |
|  | Auto | Streptococcus | 43397.22483 | -4.44928326 | 7.20E-12 | 9.21E-10 | Auto |
|  |  | Capnocytophaga | 2316.079586 | -4.05182600 | 7.13E-08 | 2.28E-06 | Auto |
|  |  | Oscillospira | 6.384047546 | -3.50403703 | 4.53E-08 | 2.28E-06 | Auto |
|  |  | Granulicatella | 484.3731939 | -3.96381873 | 5.36E-08 | 2.28E-06 | Auto |
|  |  | Haemophilus | 3740.626766 | -3.32053991 | 1.02E-06 | 2.61E-05 | Auto |
|  |  | Peptoniphilus | 6.583738276 | -2.99158471 | 3.23E-06 | 6.88E-05 | Auto |
|  |  | Paludibacter | 337.9137641 | -3.71025981 | 5.38E-06 | 9.85E-05 | Auto |
|  |  | Campylobacter | 10155.05796 | -3.28639244 | 6.73E-06 | 0.000107632 | Auto |
|  |  | Lactobacillus | 53.83936777 | -3.11344099 | 8.76E-06 | 0.000124596 | Auto |
|  |  | Actinomyces | 3047.078198 | -3.15886650 | 2.24E-05 | 0.000286287 | Auto |
|  |  | Megasphaera | 368.1254938 | -3.20525499 | 3.94E-05 | 0.000458055 | Auto |

Significantly different genera identified between normal stomach samples and PPI, autoimmune atrophic gastritis, *H. pylori*-induced atrophic gastritis and *H. pylori* gastritis. The most significant species are identified at the top. Differential expression analysis based on the Negative Binomial (Gamma-Poisson) distribution and were corrected for multiple comparisons. * indicates a genus no longer significant when *H. pylori* was removed from the analysis.
