# Supplementary material for: Comparison of the human gastric microbiota in hypochlorhydric states arising as a result of Helicobacter pylori-induced atrophic gastritis, autoimmune atrophic gastritis and proton pump inhibitor use
Source: PLoS Pathog. 2017 Nov 2;13(11):e1006653. doi: 10.1371/journal.ppat.1006653 (PMC5667734; doi:10.1371/journal.ppat.1006653)
Supplement: S2 Table — (A)Stable bacterial populations and correlations in PPI patients compared to other groups (if the correlation between two genera were consistently positive or negative in different groups). PPI versus H. pylori-induced atrophic gastritis in table S2B. No significant comparisons were found between PPI and autoimmune atrophic gastritis groups. (B) Stable bacterial populations and correlations in H. pylori-induced atrophic gastritis patients compared to other groups. (DOCX) [file ppat.1006653.s007.docx]

**Table S2A**. Stable bacterial populations and correlations in PPI compared to other groups

|  |  | **taxa1** | **taxa2** | **PPI(R)** | **PPI(Adj.P)** | **Control(R)** | **Control(Adj.P)** |
| --- | --- | --- | --- | --- | --- | --- | --- |
| Controls | 1 | Geodermatophilus | Paracoccus | 0.5662856 | 0.016819623 | 0.5773502 | 0.013548223 |
|  | 2 | Skermanella | Deinococcus | 0.5717373 | 0.016143586 | 0.6271277 | 0.006847234 |
|  | 3 | Kocuria | Skermanella | 0.8104762 | 2.58E-05 | 0.6871295 | 0.00246191 |
|  | 4 | Microbacterium | Methylobacterium | 0.7091112 | 0.002238097 | 0.5353033 | 0.018005957 |
|  | 5 | [Prevotella] | Haemophilus | 0.5883363 | 0.013964266 | 0.5259999 | 0.020246485 |
|  | 6 | Micrococcus | Geodermatophilus | 0.5289436 | 0.023766495 | 0.6625386 | 0.003973611 |
|  | 7 | Prevotella | Bulleidia | 0.5037175 | 0.028728962 | 0.5098105 | 0.022296511 |
|  | 8 | __Unknowns__ | Skermanella | 0.7103038 | 0.002238097 | 0.7717823 | 0.00073392 |
|  | 9 | __Unknowns__ | Prevotella | -0.6245059 | 0.025795752 | -0.863704 | 1.40E-05 |
| Hp Gastritis |  | **taxa1** | **taxa2** | **PPI(R)** | **PPI(Adj.P)** | **HPGast(R)** | **HPGast(Adj.P)** |
|  | 1 | Kocuria | Skermanella | 0.8104762 | 2.58E-05 | 0.7105461 | 0.00031618 |
|  | 2 | __Unknowns__ | Skermanella | 0.7103038 | 0.002238097 | 0.5683338 | 0.005786707 |

(A) Stable bacterial populations and correlations in PPI patients compared to other groups (if the correlation between two genera were consistently positive or negative in different groups). PPI versus *H. pylori*-induced atrophic gastritis in table S2B. No significant comparisons were found between PPI and autoimmune atrophic gastritis groups.

**Table S2B**. Stable bacterial populations and correlations in *H. pylori* atrophy compared to other groups

|  |  | **taxa1** | **taxa2** | **Atrophy(R)** | **Atrophy(Adj.P)** | **Control(R)** | **Control(Adj.P)** |
| --- | --- | --- | --- | --- | --- | --- | --- |
| Control | 1 | Paracoccus | Truepera | 0.613697 | 0.003116 | 0.589029 | 0.011422 |
|  | 2 | Brachybacterium | Truepera | 0.672593 | 0.001043 | 0.699917 | 0.002462 |
|  | 3 | Micrococcus | Skermanella | 0.625484 | 0.002534 | 0.510548 | 0.022297 |
|  | 4 | Corynebacterium | Skermanella | 0.549504 | 0.008859 | 0.523667 | 0.020641 |
|  | 5 | Deinococcus | Corynebacterium | 0.678279 | 0.000929 | 0.593092 | 0.011136 |
|  | 6 | Haemophilus | Agrobacterium | 0.514414 | 0.012555 | 0.512406 | 0.021989 |
|  | 7 | Haemophilus | [Prevotella] | 0.555701 | 0.007976 | 0.526 | 0.020246 |
|  | 8 | Mycoplasma | TG5 | 0.722999 | 0.000409 | 0.616347 | 0.008008 |
|  | 9 | Kocuria | Skermanella | 0.850805 | 3.74E-07 | 0.68713 | 0.002462 |
|  | 10 | Kocuria | Corynebacterium | 0.717575 | 0.000477 | 0.715449 | 0.002462 |
| PPI |  | **taxa1** | **taxa2** | **Atrophy(R)** | **Atrophy(Adj.P)** | **PPI(R)** | **PPI(Adj.P)** |
|  | 1 | Cloacibacterium | Capnocytophaga | 0.651547 | 0.001632 | 0.570736 | 0.016144 |
|  | 2 | Sphingomonas | Skermanella | 0.543353 | 0.009532 | 0.712055 | 0.002238 |
|  | 3 | Rhodococcus | Actinomyces | 0.520292 | 0.011632 | 0.550482 | 0.019776 |
|  | 4 | Rhizobium | Delftia | 0.722999 | 0.000409 | 0.544862 | 0.019776 |
|  | 5 | Prevotella | [Prevotella] | 0.773282 | 0.000105 | 0.6445 | 0.00627 |
|  | 6 | Fusobacterium | [Prevotella] | 0.587715 | 0.004808 | 0.839116 | 1.22E-05 |
|  | 7 | Veillonella | Capnocytophaga | 0.559326 | 0.007573 | 0.507724 | 0.028408 |
|  | 8 | Veillonella | Prevotella | 0.859474 | 1.59E-06 | 0.827992 | 1.61E-05 |
|  | 9 | Veillonella | Campylobacter | 0.816683 | 1.90E-05 | 0.516044 | 0.026515 |
|  | 10 | Haemophilus | [Prevotella] | 0.555701 | 0.007976 | 0.588336 | 0.013964 |
|  | 11 | Streptococcus | Prevotella | 0.674698 | 0.000998 | 0.597186 | 0.012233 |
|  | 12 | Kocuria | Skermanella | 0.850805 | 3.74E-07 | 0.810476 | 2.58E-05 |
| Hp Gastritis | | **taxa1** | **taxa2** | **Atrophy(R)** | **Atrophy(Adj.P)** | **HPGast(R)** | **HPGast(Adj.P)** |
|  | 1 | Kocuria | Skermanella | 0.850805 | 3.74E-07 | 0.710546 | 0.000316 |
|  | 2 | Kocuria | Helicobacter | -0.82327 | 1.87E-06 | -0.73367 | 0.000102 |
| Autoimmune atrophy | | **taxa1** | **taxa2** | **Atrophy(R)** | **Atrophy(Adj.P)** | **Auto(R)** | **Auto(Adj.P)** |
|  | 1 | Stenotrophomonas | Delftia | 0.722999 | 0.000409 | 1 | 0 |

(B) Stable bacterial populations and correlations in *H. pylori*-induced atrophic gastritis patients compared to other groups.
