# Supplementary material for: Comparison of the human gastric microbiota in hypochlorhydric states arising as a result of Helicobacter pylori-induced atrophic gastritis, autoimmune atrophic gastritis and proton pump inhibitor use
Source: PLoS Pathog. 2017 Nov 2;13(11):e1006653. doi: 10.1371/journal.ppat.1006653 (PMC5667734; doi:10.1371/journal.ppat.1006653)
Supplement: S3 Table — (DOCX) [file ppat.1006653.s008.docx]

**Table S3**. Most significant pathways for each group comparison

| Group Comparison | | Pathway | Group with increased representation |
| --- | --- | --- | --- |
| Control | PPI | n/a | n/a |
|  | Hp Gastritis | Homoserine dehydrogenase  Shikimate dehydrogenase  Alcohol dehydrogenase  (R,R)−butanediol dehydrogenase / diacetyl reductase  Glycerol dehydrogenase  D−arabinitol 4−dehydrogenase  L−iditol 2−dehydrogenase  Mannitol−1−phosphate 5−dehydrogenase  Myo−inositol 2−dehydrogenase  UDPglucose 6−dehydrogenase  Histidinol dehydrogenase | Hp Gastritis  Hp Gastritis  Control  Control  Control  Control  Control  Control  Control  Control  Control |
|  | Hp Atrophy | Ketol−acid reductoisomerase  Glycolate oxidase  Formate dehydrogenase, beta subunit  Alanine dehydrogenase  UDPglucose 6−dehydrogenase  Malate dehydrogenase, (oxaloacetate−decarboxylating)  Tagaturonate reductase  Succinate dehydrogenase flavoprotein subunit, iron−sulfur subunit, cytochrome b556 subunit  Glutamate synthase (NADPH/NADH) small chain  L-aspartate oxidase | Hp Atrophy  Hp Atrophy  Hp Atrophy  Hp Atrophy  Control  Control  Control  Control  Control  Control |
|  | Autoimmune Atrophy | Hydroxymethylglutaryl−CoA reductase  Alpha−glycerophosphate oxidase  Pyruvate oxidase  Phenylalanine dehydrogenase  Trimethylamine dehydrogenase  Ribonucleoside−diphosphate reductase beta chain  Arsenate reductase  Betaine−homocysteine S−methyltransferase  L−aspartate oxidase  5−methyltetrahydrofolate−−homocysteine methyltransferase | Autoimmune  Autoimmune  Autoimmune  Autoimmune  Autoimmune  Autoimmune  Autoimmune  Autoimmune  Control  Control |
| PPI | Hp Gastritis | Homoserine dehydrogenase  Shikimate dehydrogenase  Alcohol dehydrogenase  (R,R)−butanediol dehydrogenase / diacetyl reductase  Glycerol dehydrogenase  D−arabinitol 4−dehydrogenase  L−iditol 2−dehydrogenase  Mannitol−1−phosphate 5−dehydrogenase  Myo−inositol 2−dehydrogenase  UDPglucose 6−dehydrogenase  Histidinol dehydrogenase | HP Gastritis  HP Gastritis  PPI  PPI  PPI  PPI  PPI  PPI  PPI  PPI  PPI |
|  | Hp Atrophy | Ketol−acid reductoisomerase  Formate dehydrogenase, beta subunit  D−lactate dehydrogenase (cytochrome)  Pyruvate ferredoxin oxidoreductase, alpha subunit  Acetyl−CoA decarbonylase/synthase complex subunit epsilon  Fumarate reductase flavoprotein subunit  UDPglucose 6−dehydrogenase  Malate dehydrogenase, (oxaloacetate−decarboxylating)  Succinate dehydrogenase flavoprotein subunit, iron−sulfur subunit, cytochrome b556 subunit  Glutamate synthase (NADPH/NADH) small chain  3−isopropylmalate dehydrogenase | Hp Atrophy  Hp Atrophy  Hp Atrophy  Hp Atrophy  Hp Atrophy  Hp Atrophy  PPI  PPI  PPI  PPI  PPI |
|  | Autoimmune Atrophy | Ketol−acid reductoisomerase  D−lactate dehydrogenase (cytochrome)  Formate dehydrogenase, beta subunit  Pyruvate ferredoxin oxidoreductase, alpha, beta, delta, gamma subunits  Acetyl−CoA decarbonylase/synthase complex subunit epsilon  Fumarate reductase flavoprotein subunit  UDPglucose 6−dehydrogenase  Malate dehydrogenase (oxaloacetate−decarboxylating)  3−isopropylmalate dehydrogenase  Succinate dehydrogenase iron−sulfur subunit | Autoimmune  Autoimmune  Autoimmune  Autoimmune  Autoimmune  Autoimmune  PPI  PPI  PPI  PPI |
| Hp Gastritis | Hp Atrophy | Alcohol dehydrogenase, (NADP+)  (R-R)-butanediol dehydrogenase / diacetyl reductase  Glycerol dehydrogenase  D−arabinitol 4−dehydrogenase  L−iditol 2−dehydrogenase  Myo−inositol 2−dehydrogenase  Histidinol dehydrogenase  Glyoxylate reductase  L−lactate dehydrogenase  Glycerate dehydrogenase | Hp Atrophy  Hp Atrophy  Hp Atrophy  Hp Atrophy  Hp Atrophy  Hp Atrophy  Hp Atrophy  Hp Atrophy  Hp Atrophy  Hp Gastritis |
|  | Autoimmune Atrophy | Alcohol dehydrogenase  (R,R)−butanediol dehydrogenase / diacetyl reductase  Glycerol dehydrogenase  D−arabinitol 4−dehydrogenase  L−iditol 2−dehydrogenase  Mannitol−1−phosphate 5−dehydrogenase  Myo−inositol 2−dehydrogenase  UDPglucose 6−dehydrogenase  Homoserine dehydrogenase  Shikimate dehydrogenase | Autoimmune  Autoimmune  Autoimmune  Autoimmune  Autoimmune  Autoimmune  Autoimmune  Autoimmune  Hp Gastritis  Hp Gastritis |
| Hp Atrophy | Autoimmune Atrophy | Glycolate oxidase  D−lactate dehydrogenase (cytochrome)  3−hydroxyisobutyrate dehydrogenase  Glucose−6−phosphate 1−dehydrogenase  D−amino−acid dehydrogenase  Hydroxymethylglutaryl−CoA reductase  Alpha−glycerophosphate oxidase  Glyceraldehyde−3−phosphate dehydrogenase (NADP)  Pyruvate oxidase  Dihydrofolate reductase  Trimethylamine dehydrogenase | Hp Atrophy  Hp Atrophy  Hp Atrophy  Hp Atrophy  Hp Atrophy  Autoimmune  Autoimmune  Autoimmune  Autoimmune  Autoimmune  Autoimmune |
