# Supplementary material for: Comparison of the human gastric microbiota in hypochlorhydric states arising as a result of Helicobacter pylori-induced atrophic gastritis, autoimmune atrophic gastritis and proton pump inhibitor use
Source: PLoS Pathog. 2017 Nov 2;13(11):e1006653. doi: 10.1371/journal.ppat.1006653 (PMC5667734; doi:10.1371/journal.ppat.1006653)
Supplement: S4 Table — (A) Significant bacterial species identified between H. pylori atrophic gastritis and H. pylori gastritis. The most significant species are identified at the top. Differential expression analysis based on the Negative Binomial (Gamma-Poisson) distribution. Streptococcus identified by BLAST as S. mitis with 98% coverage, 99% identity and Neisseria mucosa had 98% coverage and 100% identity. None of these OTUs remained significant when H. pylori was removed from the analysis. (B) Significant bacterial genera identified between autoimmune atrophic gastritis and H. pylori-induced atrophic gastritis. The most significant species are identified at the top. Differential expression analysis based on the Negative Binomial (Gamma-Poisson) distribution. NB when H. pylori was removed from the analysis these genera remained significant, with an additional genus Desulfobulbus also reaching significance. (DOCX) [file ppat.1006653.s009.docx]

**Table S4A.** Significantly different OTUs identified between *H. pylori* gastritis and *H. pylori* atrophy

| **OTU** | **baseMean** | **log2 Fold Change** | **P value** | **P value adjusted** | **Upregulated** |
| --- | --- | --- | --- | --- | --- |
| OTU4129 pylori | 32.2692244 | -3.75862637 | 2.01E-12 | 2.71E-09 | Atrophy |
| OTU7300 pylori | 53.3457099 | -3.47126369 | 2.05E-11 | 1.38E-08 | Atrophy |
| OTU2594 pylori | 55.5859339 | -3.14220741 | 3.73E-10 | 1.67E-07 | Atrophy |
| OTU1431 pylori | 7.68077131 | -2.79190038 | 1.14E-08 | 3.82E-06 | Atrophy |
| OTU9201 pylori | 7.19698133 | 2.5113443 | 8.06E-08 | 2.17E-05 | HPGast |
| OTU3164 pylori | 7.54736188 | 2.536829207 | 9.85E-08 | 2.21E-05 | HPGast |
| OTU7547 pylori | 5.81973568 | -2.53005214 | 1.15E-07 | 2.21E-05 | Atrophy |
| OTU6868 pylori | 5.41957740 | -2.45293771 | 2.48E-07 | 4.17E-05 | Atrophy |
| OTU8956 Streptococcus (mitis) | 6.17921709 | -2.13009995 | 2.32E-06 | 0.0003474 | Atrophy |
| OTU7757 subflava (Neisseria mucosa) | 12.4036588 | -2.14453347 | 7.22E-06 | 0.0009707 | Atrophy |

(A) Significant bacterial species identified between *H. pylori* atrophic gastritis and *H. pylori* gastritis. The most significant species are identified at the top. Differential expression analysis based on the Negative Binomial (Gamma-Poisson) distribution. *Streptococcus* identified by BLAST as *S. mitis* with 98% coverage, 99% identity and *Neisseria mucosa* had 98% coverage and 100% identity. None of these OTUs remained significant when *H. pylori* was removed from the analysis.

**Table S4B.** Significantly different genera identified between *H. pylori* atrophy and autoimmune atrophy

| **Genus** | **baseMean** | **log2 Fold Change** | **P value** | **P value adjusted** | **Upregulated** |
| --- | --- | --- | --- | --- | --- |
| Tannerella | 172.8084262 | 5.879436599 | 1.98E-16 | 2.80E-14 | Auto |
| Dorea | 74.10482086 | 5.518494439 | 6.49E-16 | 4.58E-14 | Auto |
| Oribacterium | 46.80824501 | 4.998665381 | 2.25E-13 | 1.06E-11 | Auto |
| Actinomyces | 2398.232829 | 6.136922152 | 3.63E-13 | 1.28E-11 | Auto |
| Megasphaera | 299.0391117 | 5.764097274 | 1.29E-12 | 2.60E-11 | Auto |
| Granulicatella | 412.2028266 | 5.70908338 | 1.26E-12 | 2.60E-11 | Auto |
| Paludibacter | 286.2808811 | 6.278796323 | 1.02E-12 | 2.60E-11 | Auto |
| Streptococcus | 37706.79939 | 5.833147862 | 2.33E-12 | 4.11E-11 | Auto |
| Catonella | 35.43357819 | 4.843339079 | 2.33E-11 | 3.51E-10 | Auto |
| Pseudomonas | 105.7330891 | 5.04178473 | 2.49E-11 | 3.51E-10 | Auto |
| Moryella | 89.14744396 | 4.842033053 | 7.00E-11 | 8.97E-10 | Auto |
| Mycoplasma | 21.45873199 | 4.5942512 | 1.83E-10 | 2.15E-09 | Auto |
| Veillonella | 1672.123902 | 4.807876037 | 7.11E-10 | 7.71E-09 | Auto |
| Campylobacter | 8271.513023 | 5.326831302 | 1.60E-09 | 1.61E-08 | Auto |
| Fusobacterium | 3403.134689 | 5.000555712 | 1.91E-09 | 1.79E-08 | Auto |
| Parvimonas | 66.34218906 | 4.47369972 | 5.81E-09 | 5.12E-08 | Auto |
| Haemophilus | 3107.566051 | 4.967639501 | 6.39E-09 | 5.30E-08 | Auto |
| Atopobium | 18.99679631 | 3.74200124 | 1.22E-08 | 9.56E-08 | Auto |
| Prevotella | 2042.729123 | 4.695405549 | 1.34E-08 | 9.96E-08 | Auto |
| Capnocytophaga | 2021.939473 | 5.241690623 | 1.44E-08 | 1.01E-07 | Auto |
| Oscillospira | 5.95173881 | 3.617220619 | 1.83E-08 | 1.23E-07 | Auto |
| Prevotella | 10156.01781 | 4.603215995 | 4.70E-08 | 3.01E-07 | Auto |
| Selenomonas | 196.4803129 | 4.437002874 | 5.48E-08 | 3.36E-07 | Auto |
| Treponema | 134.4817054 | 4.187925289 | 6.17E-08 | 3.63E-07 | Auto |
| Bulleidia | 38.28508589 | 3.989951184 | 9.62E-08 | 5.43E-07 | Auto |
| Leuconostoc | 5.936742635 | 3.361224441 | 2.57E-07 | 1.39E-06 | Auto |
| Helicobacter | 67765.73481 | -4.96377804 | 5.96E-07 | 3.11E-06 | Atrophy |
| Leptotrichia | 656.2439409 | 3.905149229 | 3.84E-06 | 1.93E-05 | Auto |
| Spirosoma | 4.785551673 | 2.608338443 | 4.83E-06 | 2.35E-05 | Auto |
| Eikenella | 3.162343133 | 2.411462483 | 5.25E-06 | 2.47E-05 | Auto |
| Peptoniphilus | 6.318625439 | 2.850371054 | 1.03E-05 | 4.68E-05 | Auto |
| Clostridium | 5.466915388 | 2.590743885 | 1.63E-05 | 7.17E-05 | Auto |
| Peptococcus | 3.176608962 | 2.46875735 | 4.85E-05 | 0.000207106 | Auto |
| Aggregatibacter | 57.68794101 | 3.074235154 | 0.000117738 | 0.000488265 | Auto |
| Neisseria | 2822.253115 | 3.483123532 | 0.000130346 | 0.000525108 | Auto |
| Acidovorax | 2.38241871 | 1.913747107 | 0.000180267 | 0.000706045 | Auto |

(B) Significant bacterial genera identified between autoimmune atrophic gastritis and *H. pylori*-induced atrophic gastritis. The most significant species are identified at the top. Differential expression analysis based on the Negative Binomial (Gamma-Poisson) distribution. NB when *H. pylori* was removed from the analysis these genera remained significant, with an additional genus Desulfobulbus also reaching significance.
